# Advancing standards for bioinformatics activities: persistence, reproducibility, disambiguation and Minimum Information About a Bioinformatics investigation (MIABi)

**DOI:** 10.1186/1471-2164-11-S4-S27

**Published:** 2010-12-02

**Authors:** Tin Wee Tan, Joo Chuan Tong, Asif M Khan, Mark de Silva, Kuan Siong Lim, Shoba Ranganathan

**Affiliations:** 1 Department of Biochemistry, Yong Loo Lin School of Medicine, National University of Singapore, 8 Medical Drive, Singapore 117597; 2 Institute for Infocomm Research, 1 Fusionopolis Way, #21-01 Connexis South Tower, Singapore 138632; 3 Department of Pharmacology and Molecular Sciences, The Johns Hopkins University School of Medicine, Maryland MD21205, USA; 4 Department of Chemistry and Biomolecular Sciences and ARC Centre of Excellence in Bioinformatics, Macquarie University, Sydney NSW 2109, Australia

## Abstract

The 2010 International Conference on Bioinformatics, InCoB2010, which is the annual conference of the Asia-Pacific Bioinformatics Network (APBioNet) has agreed to publish conference papers in compliance with the proposed Minimum Information about a Bioinformatics investigation (MIABi), proposed in June 2009. Authors of the conference supplements in BMC Bioinformatics, BMC Genomics and Immunome Research have consented to cooperate in this process, which will include the procedures described herein, where appropriate, to ensure data and software persistence and perpetuity, database and resource re-instantiability and reproducibility of results, author and contributor identity disambiguation and MIABi-compliance. Wherever possible, datasets and databases will be submitted to depositories with standardized terminologies. As standards are evolving, this process is intended as a prelude to the 100 BioDatabases (BioDB100) initiative whereby APBioNet collaborators will contribute exemplar databases to demonstrate the feasibility of standards-compliance and participate in refining the process for peer-review of such publications and validation of scientific claims and standards compliance. This testbed represents another step in advancing standards-based processes in the bioinformatics community which is essential to the growing interoperability of biological data, information, knowledge and computational resources.

## **Background**

Over the past decade the volume of bioinformatics publications has grown tremendously. Within the scientific community, there have been concerns about disappearing databases, lack of interoperability, incomplete disclosure, and general quality and integrity issues. Efforts have been made to promote uniqueness and universality in standardized identifiers including Life Science Identifiers (LSID) [1], Digital Object Identifiers (DOI) [2] and CrossRef [3], author and contributor identifiers [4] (e.g. ResearcherID [5], Scopus Author Identifier [6], Open Researcher & Contributor ID [7]); in checklists of minimum information reporting (such as the MIBBI project [8]); and in the use of standard nomenclature, controlled vocabularies and ontologies [9]. The ultimate goal would be for the community to achieve a systematically organized, universally adopted and disciplined approach in building and organizing the corpus of biological knowledge that is accurate, reliable, trustworthy, consistent and persistent. The entire information infrastructure endorsed and used by the community should be universally accessible, wholly interoperable, secure, robust, sustainable in perpetuity, backward compatible and where possible, future proofed.

Just like the Internet, where open standards backed by implementable code and use-case exemplars are universally adopted, all actors and stakeholders in the process of scientific discovery and publication must be involved if we are to achieve bioinformatics standards of comparable universality. In our effort to advance standards for bioinformatics activities, we have assembled a set of publishers, editors, reviewers, authors, contributors, database and resource administrators, programmers and scientists who are involved in our annual International Conference on Bioinformatics and the publication of our conference supplements, as an exemplar of how such a process for standardization can take place. Through this multi-stakeholder effort, we hope that the lessons learnt will be able to shed light on how we can plan and coordinate a systematic approach and evaluate them for possible extension to a wider community. The aspects tested in this process are limited to the following, which we believe is sufficiently wide enough for facilitating a larger effort to build 100 biological and bioinformatics databases (BioDB100) which are standards-compliant:

a. Data and software persistence and the basis for perpetuity [10]

b. Re-instantiability and reproducibility [11]

c. Author and Contributor Identity Disambiguation [12]

d. Minimum Information about a Bioinformatics investigation (MIABi) as described in this report and as harmonized with initiatives in MIBBI [8] and those of the International Society for Biocuration (ISB) [13], specifically BioDBcore [14].

With this platform of standards-compliant databases, we hope that it will lay the groundwork for studying how we can implement increasingly standardized terminology, controlled vocabulary, standardized ontologies and infrastructural and informational interoperability, such as use of international computational grids [15] and cloud computing as backend computing resources for the maintenance and sustainability of knowledge resources of ever-increasing sophistication.

### **Data and software repository**

Software, datasets and databases described in the International Conference on Bioinformatics, InCoB2010 published in this conference supplement issue are required to be deposited in distributed repositories which have sufficient collective guarantee of perpetuity. As much as all open source publicly available papers can be accessed long after the publication date, the corresponding data, datasets and software described should be just as easily accessible for future researchers to reproduce, verify and validate the claims and findings or otherwise, and hopefully to improve on them and advance the field. A deposition of material corresponding to each publication with version control, unique identifiers, adequate descriptors and metadata, should be managed and properly curated, as part and parcel of the publication process. Currently many journals have a section called "supplementary material" where additional information can be tagged along with the main publication. This material, however, is generally unstructured or little effort is made to establish metadata to describe the content.

We propose that when databases are described in a publication, a copy should be frozen, version-labelled and dated for reference. Any subsequent updates with fully described new features or errata can also be added for supplementation of the initial deposited material. Should the corresponding live database referenced in the publication be discontinued, the original database must be re-instantiable upon request by any reader wishing to review the work, without undue administrative, operational or technical barriers, and wherever possible, future-proofed. Likewise, any Web service with its computational or database backends should also be similarly deposited in a re-instantiable form. A prototype system [10] is currently being worked on to implement as many stakeholder requests for features to be added to the system to facilitate ease of deposition.

Deposited material with accompanying metadata will be compliant with minimum information standards as described below, as well as harmonized with those of other initiatives, such as those in the MIBBI project [8] and that of the International Biocuration Society [13], its BioDBCore specification [14] and others.

### **Re-instantiability and reproducibility**

To facilitate any research work that might require technical assistance for re-instantiability, we have developed a virtual machine (VM) platform that can be used on any current operating system to replay back an operating system image of the entire database, software application or Web service [11]. This platform is based on Slax, a LiveCD-based Slackware distribution of the Linux operating system which we have configured for bioinformatics (BioSlax). Based on our success of this re-instantiable platform on VMs (such as VMware's virtual machine platform) running on any current operating systems, or on full hardware virtualization of cloud computing platforms (such as the open source Citrix Xen® Hypervisor for virtualization of operating systems across a wide range of CPU architectures), we envisage the possibility of full compliance to the requirement of re-instantiability.

As more such stable, standards-based platforms of different operating systems can be achieved, on which our authors can wrap their online services in re-instantiable forms that suit their specific requirements, our community can progress to a level of peer-review where the reviewer does not need to take the data and the research procedures solely on good faith. Our reviewers in the peer-review process can selectively or comprehensively take author-submitted data and subject them to as much rigour as they wish in testing the validity of claims made by the authors. Unlike wet-laboratory experiments, where reviewers use their best judgement to consider whether the experimental disclosure is sufficient for reproduction by any personal skilled in the art, and where experiments require great time and expense to be repeated during the review process, the rigour of the review process for bioinformatics investigations can reach a level where the author's *in silico* experiments can be selectively or completely reproduced, depending on the computational power and resources available to the reviewer. In this way, the veracity of the claims can be tested and any queries be raised before the paper can be approved for publication. Moreover, any doubtable claims can be refuted or rebutted before publication. Any software coding errors can be detected earlier, and any database errors fixed before public release.

For example, suppose a piece of software is described that runs on a particular operating system. The author might be required to provide at least one form of the software compliant with the re-instantiable operating system, without requiring the end user or the reviewer to carry out complex installation procedures and face installation problems with dependencies on other components of software which need to be pre-installed before the software to be published can work well. For future proofing, the author may be required to wrap the software together with a compatible operating system, fully configured to be re-instantiable on a virtual machine that may be emulated on a wider set of compatible operating systems and hardware.

### **Supporting grid and cloud computing infrastructure**

To support such re-instantiable operating systems containing datasets, databases, web-resources, pre-compiled software applications, and version control of each deposited digital object, as well as the metadata describing what is stored, large data storage platforms have to be procured. Uniquely labeled versions and copies must be widely distributed to ensure minimized risk of data loss and to avoid confusion. To support the ability of reviewers to assess the assertions of authors and to validate the results pre-publication, and for readers to reproduce the *in silico* experiments described by the authors, high performance computing infrastructure needs to be in place where datasets and software applications are co-located in a grid infrastructure or virtualized in a cloud computing environment. Moreover, there is a trend to document the scientific process for *in silico* e-science and encapsulate multiple steps of a long workflow into concise machine readable workflow integration systems including popular efforts such as Taverna [16] and Galaxy [17]. These workflows together with their orchestration engines, need grid and cloud computing infrastructure in order to be used by the publication process which we are implementing in the MIABi effort and the BioDB100 testbed. We are currently discussing with colleagues in the virtual organizations of OpenScienceGrid.org and EUAsiaGrid for e-infrastructure support. The issues faced in such a test implementation will be documented and analysed for its feasibility for wider scale deployment.

### **Author disambiguation**

Author names, particularly Asian author names, for various reasons, have a high degree of convergence. Higher populations of people using a smaller set of common family or surnames, often result in author name ambiguities. Despite the emergence of author identifier systems such as Researcher ID from Thomson Reuters [5] and Scopus Author ID from Elsevier [6], Open Researcher and Contributor ID (ORCID) [7] and many others, universal, unique and unambiguous author identifier for author disambiguation remains elusive [18].

For our BioDB100 testbed, we propose that our authors use a self-editable author identifier with a protocol to equivalence synonymous identifiers on a neutral non-publisher-specific platform. Where an author may end up with several author identifiers on different systems, we provide an equivalencing mechanism, making all of them synonymous, tracing back different author identifiers to its unique owner. Any duplicate claims can also undergo a dispute resolution mechanism to resolve competing claims of ownership over identifiers or the papers associated with author identifiers. Erroneous author identifier labels, tagged to a publication, can also lend themselves to a protocol for de-convolution. Two or more authors with exactly the same name label on different publications can be disambiguated. The same author with different name labels on a publication, due to printing errors, or due to transliteration or translational ambiguity, can also apply disambiguation using such a system. A prototype system [3] (based on the distributed Internet domain name system) is currently being established with user-friendly interfaces to test usability and to develop machine-resolvable author disambiguation requests. By validating the corresponding authors in our conference supplements and in the BioDB100 initiative, we combine the self-assertion of the author identifier system with an external validation mechanism, thus providing support for the hybrid-asserted identity model of Bilder [19].

Using this protocol, publication tags such as PubMed identifiers or DOI handles can be associated by the author directly to the set of equivalent author identifiers and author labels. There is now hope for a person who has changed names, in the case of women changing family names upon marriage, or of name order changes, for instance a fictitious Vivian George who previously used George Vivian or G. Vivian because this was the country convention in India (family or father’s or village name abbreviated into an initial), now decides to switch to V. George because he is based in the US, where last names come last. There is also a linking mechanism for authors who have changed fields, as well as those who are victims of errors of author identifier systems, such as those giving the same author more than one identifier because of the best guess attempt by an automated system that failed to determine that two papers were published by the same person, and because of time, geographic distance, differences in affiliation or disparate fields of study, they were given two different unique author identifiers.

### **Minimum Information about a Bioinformatics Investigation (MIABi)**

In 2001, efforts to compare microarray experiments eventually led to a standardization known as the Minimum Information about Microarray Experiments (MIAME) [20]. Since then, many other minimum information standards have appeared, now coordinated by the project on Minimum Information about a Biological or Biomedical Investigation (MIBBI) [8]. We would like to draw the community’s attention to the Minimum Information about a Bioinformatics investigation (MIABi) initiative, which specifies, through a series of documentation modules, the minimum information that should be provided for a bioinformatics investigation. Developed through a joint effort by the Asia-Pacific Bioinformatics Network (APBioNet) (http://www.apbionet.org/) and the wider bioinformatics community [21], the MIABi initiative arises from a response to the growing need for transparency, provenance and scientific reproducibility amongst the bioinformatics and computational biology community. Currently, it is increasingly common for computational tools to be applied to ever larger datasets in order to generate output with little commensurate effort to review objectively the quality of the process undertaken, or the quality of the input data, analytical process, or the conclusions drawn. MIABi plays a key role in at least one of the steps taken to prevent any decline in the overall value of the scientific publications in bioinformatics and computational biology. It also aims at “minimizing the reporting requirements while maximizing the information available to those interpreting the results” of a bioinformatics database, resource, software application or algorithm, analysis or *in silico* experiment.

**Figure 1:**
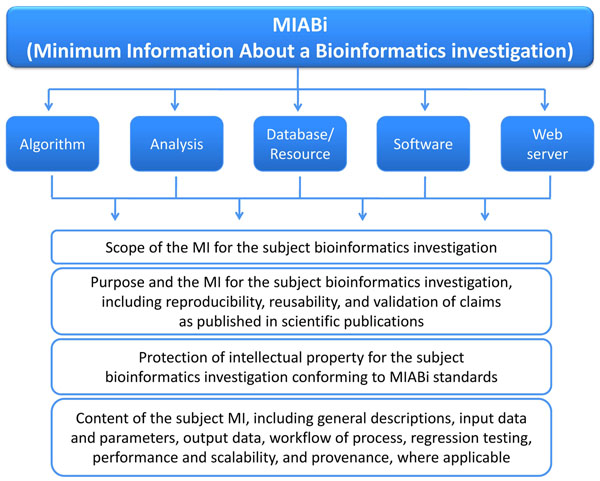
**Content overview in MIABi**. The sections and sub-sections of the full MIABi document are set out.

**Figure 2:**
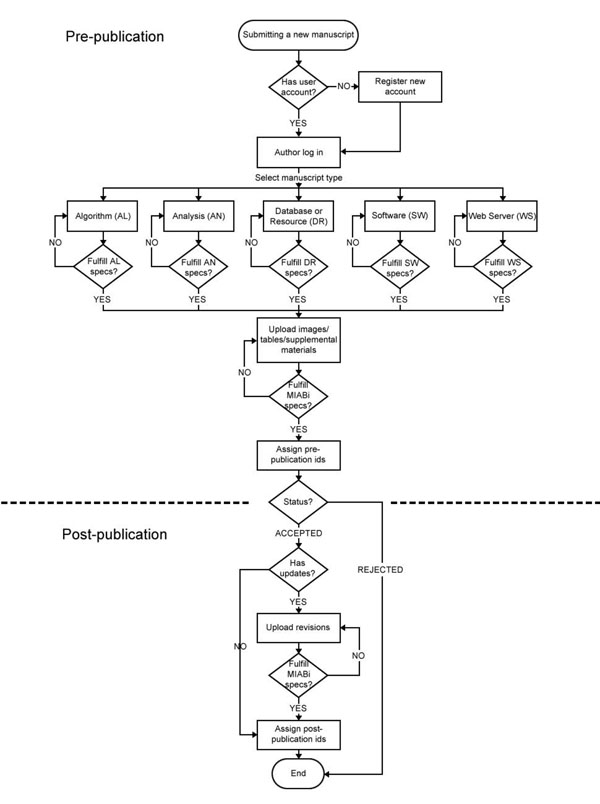
**The MIABi workflow.** Pre-publication and post-publications sections of the workflow are elaborated.

The guidelines (see Additional File 1) cover the Minimum Information About a Bioinformatics i) algorithm, ii) analysis, iii) database or resource, iv) software, and v) Web server. The MIABi scope and workflow is shown in Figures 1 and 2. This MIABi compliance will require, firstly, for authors to be issued with unique author identifiers (http://aid.apbionet.org/) for identity disambiguation and accountability purposes. Authors with multiple identifiers issued by various publishers (e.g. Scopus author ID, ResearcherID) as mentioned above, can now be resolved to a unique individual through cross-referencing and synonymization of these identifiers. Secondly, it will require deposition of scientific datasets through a central portal (e.g. http://docid.apbionet.org/) for persistence, provenance, accessibility and reproducibility. All databases, datasets and codes cited in papers published through such processes may be mandated to be archived in this way, supported by distributed repository nodes, such as that of the Asian Bioinformation Center initiative and the nascent e-Science Collaboration between Asia-Pacific and Europe (eSCAPE). Moreover, a database on a pre-configured operating system (OS) such as BioSlax (http://www.bioslax.com) can also be archived as an image and stored at such repositories. Should the original database server be unavailable, the database-OS image can be dynamically re-instantiated on demand via a cloud computing virtualized platform. Other activities in bioinformatics such as phylogenetic analysis, which has already been initiated by the Minimum Information About a Phylogenetic Analysis (MIAPA) group [22] or for the curation of databases [14], which is being initiatied by the International Society for Biocuration [13], may be referenced, integrated or unified, wherever appropriate. By following the general trend in the bioscience community for the standardization of reporting, MIABi will be registered with the MIBBI Project [8], so as to promote and coordinate the development, management and harmonization of Minimum Information (MI) specifications from across the biological and biomedical sciences.

In keeping with the acceptable processes in the development and growing adoption of MI standards, MIABi aims to be as applicable as possible to a wide range of computational technologies.

Currently, the full MIABi document is divided into five sections: i) algorithm, ii) analysis, iii) database or resource, iv) software, and v) Web server (Figure 2). Each section is subdivided as follows: 1) scope of the MI for the subject bioinformatics investigation; 2) purpose and the MI for the subject bioinformatics investigation, including reproducibility, reusability, and validation of claims as published in scientific publications; 3) protection of intellectual property for the subject bioinformatics investigation conforming to MIABi standards and 4) content of the subject MI, including general descriptions, input data and parameters, output data, workflow of process, regression testing, performance and scalability, and provenance, where applicable. For the MIABi for databases (MIABi-DB), we are working with the International Biocuration Society on harmonization of our schema with their BioDBcore checklists [14, personal communication with P Gaudet, S-A Sansone and Biocuration team members].

## **Standardization of terminologies**

At its current incarnation, MIABi will not mandate all dataset or database submissions to be fully compliant with standard nomenclature, controlled vocabularies and standard ontologies, for the practical reason that many scientific communities may not be ready to agree to the standardization process or competing standards or that there are no standard terminologies in many rapidly growing fields. Nevertheless, it is our hope that where communities are ready, they should be able to build their vocabularies, formulate their field-specific ontologies, and develop consistent processes for their updates and provide the semantic platform for building consistent terminologies that pertain to that field. Once this is done, any terminology inconsistency between fields and subfields can be addressed, and any collision of terminology de-collided or disambiguated. In a decontextualised state, similar or identical terms create confusion for reasoning engines or natural language processing. Perhaps a universal terminology deconvolution engine might help, very much like a scaled-up version of Wikipedia's disambiguation system.

## **Discussion**

Through our effort to implement MIABi standards and to test it on real world databases in the BioDB100 project, we hope that in the conceivable future, a research publication in bioinformatics will not only consist of the typical paper, but also as a matter of common practice, include deposited and quality-controlled metadata about the research process; in addition, any database, software or datasets arising from the publication shall be accessible from a globally distributed repository in a standards-compliant form, ready for reproducibility of the published results and for reusability in future research (with due consideration to the copyright owners and the authors).

From the outcome of the key issues covered in this standardization deployment project, we hope that sufficient lessons will be learnt such that we can create a community of researchers and stakeholders who are open to the standardization process, and attain a set of implementable standards and procedures. The process of developing policy and procedural protocols that derive from this endeavour will hopefully be sufficiently robust for us to test on a standards-compliant set of biological databases which the APBioNet plans to initiate as the BioDB100 project. This set of the first 100 biological databases contributed by our Asia-Pacific community and beyond, can then be used as a basis for informed debate and discussion, and may eventually serve as an exemplar towards the wider standardization of bioinformatics activities.

## **Competing interests**

The authors declare that they have no competing interests.

## Supplementary Material

Additional File 1 MIABi Reporting guidelines for a bioinformatics investigation (MIABi version 1.01). Click here for file
