# Supplementary material for: Advancing standards for bioinformatics activities: persistence, reproducibility, disambiguation and Minimum Information About a Bioinformatics investigation (MIABi)
Source: BMC Genomics. 2010 Dec 2;11(Suppl 4):S27. doi: 10.1186/1471-2164-11-S4-S27 (PMC3005918; doi:10.1186/1471-2164-11-S4-S27)
Supplement: Additional File 1 — MIABi Reporting guidelines for a bioinformatics investigation (MIABi version 1.01). [file 1471-2164-11-S4-S27-S1.pdf]

Advancing standards for bioinformatics activities: persistence, reproducibility, disambiguation and Minimum Information About a Bioinformatics investigation (MIABi)

Tin Wee Tan, Joo Chuan Tong, Asif M. Khan, Mark de Silva, Kuan Siong Lim & Shoba Ranganathan

Additional File 1:

MIABi Reporting guidelines for a bioinformatics investigation (MIABi version 1.01)

As this process is experimental, the version of the MIABi standards published herein, is open to rapid change, especially where efforts are currently in progress to harmonize these guidelines with checklists from the International BioCuration Society's BioDBCore (personal communication P. Gaudet and S.-A. Sansone) and other similar efforts. We will undertake to maintain for our authors an up-to-date version of the MIABi which they have to comply with at our online webpages

MIABi-AL (Algorithm)

Scope – MIABi-AL is the Minimum Information About a Bioinformatics ALgorithm which defines the minimum information reported for a new bioinformatics algorithm.

Purpose – MIABi-AL contains information that can allow anyone suitably skilled in the art, to reproduce the algorithm with some effort (with the exception of proprietary algorithms), to verify the veracity of the claims of the authors of such an algorithm found in the scientific publication that cites its compliance.

Protection of Intellectual Property – Where an algorithm is not intended for open source or open access, and its content, though described as far as possible in compliance to MIABi, is classified proprietary and not publicly revealed, then the MI disclosure shall include full reference to the source of these proprietary material(s) and the mechanism for access, licensing or purchase, as the case may be, in order that the claims made by the publication can be verified or validated.

Content – MIABi-AL shall contain the following minimal information:

a) General descriptions

- Date stamp (as YYYY-MM-DD)
- Responsible person (or institutional role if more appropriate); provide name, affiliation and stable contact information
- Type of algorithm
- Programming language used
- Platforms supported
- For all external software that contributed to creating the algorithm, the name, version and manufacturer
- Customizations made to that software
- Availability of that software
- Overall research process, workflow pipelines and data flow diagrams where appropriate

b) Input data

- Source of input data (e.g. from experimental information, data mining or existing datasets)
- Description of all such sources, availability, location and accessibility

- General statistics of input data, including size of dataset and nature of the data
- Data transformation and normalization technique
- File type and format

c) Input parameters

- All parameters used to create the new algorithm
- Thresholds, where available; for deriving the claims made by the algorithm

d) Output from the procedure

- Description of output
- Sequence (for identified nucleotides, peptides, or proteins)
- Quantitative scores

e) Regression testing, performance and scalability

- Description of the training and test datasets to check the validity of the claims made by the algorithm
- Description of the regression test process to verify the claims made by the algorithm

f) Provenance

- All metadata regarding the provenance of the algorithm and where they are accessible for independent enquiry, audit, review, assessment, verification and validation.

MIABi-AN (Analysis)

Scope – MIABi-AN is the Minimum Information About a Bioinformatics ANalysis which defines the minimum information reported in a data analysis publication.

Purpose – MIABi-AN contains information that can allow anyone suitably skilled in the art, to reproduce the analysis with some effort, to verify the veracity of the claims of the authors of such an analysis found in the scientific publication that cites its compliance and to evaluate the conclusions made by the authors, as derived from the results of the analysis.

Content – MIABi-AN shall contain the following minimal information.

a) General descriptions

- Date stamp (as YYYY-MM-DD)
- Responsible person (or institutional role if more appropriate); provide name, affiliation and stable contact information
- Goals and objectives
- Type of analysis (e.g. sequence analysis, predictions, simulations, models, phylogenetic analysis, biostatistical analysis)

b) Workflow of the analysis

- Type, size and source of data analyzed
- Datasets used in the analysis (to be deposited under MIABi-DR)
- Data transformation and normalization technique, where applicable
- Methodology for each step of analysis, including detailed description of each and every software or algorithm used to derive the claims made by the final results

Status – This document is likely to be elaborated into more specific documents that will define in greater detail the minimum information that is required to be disclosed for specific bioinformatics analysis, including sequence analysis, predictions, simulations, models, phylogenetic analysis, biostatistical analysis based on a

bioinformatics database or dataset, correlations, data from genomics, proteomics, mass spectrometry, structural biology, microarrays and genome-wide analysis.

MIABi-DR (Database or Resource)

Scope – MIABi-DR is the Minimum Information About a Bioinformatics Database or Resource which defines the minimum information reported for a new bioinformatics dataset, database or resource.

Purpose – MIABi-DR contains information that can allow anyone suitably skilled in the art, to reproduce the database with some effort (with the exception of proprietary databases), to verify the veracity of the claims of the authors of such a dataset, database or resource found in the scientific publication that cites its compliance.

Protection of Intellectual Property – Where a database or resource is not intended for open source or open access, and that its content, though described as far as possible in compliance to MIABi, is classified proprietary and not revealed, then the MI disclosure shall include full reference to the source of these proprietary material and the mechanism for access or licensing or purchase, as the case may be, in order that the claims made by the publication can be verified or validated.

Content – MIABi-DR shall contain the following minimal information:

a) General descriptions

- Date stamp (as YYYY-MM-DD)
- Responsible person (or institutional role if more appropriate); provide name, affiliation and stable contact information
- Type of database or resource
- Platforms supported

- For all software used in creating the database or resource, the name, version and manufacturer
- Customizations made to that software
- Availability of that software
- Location of the database or resource
- Accessibility of the database or resource, including mechanism of access (e.g. Internet)
- Frequency of updates
- Overall research process, workflow pipelines and data flow diagrams where appropriate
- Source of repository for access to the database or resource in addition to existing Internet site

b) Input data

- Source of input data (e.g. from experimental information, data mining, existing datasets or existing databases)
- Procedure of curation (manual, semi-automated or fully automated)
- Description of all such sources, availability, location and accessibility
- General statistics of input data, including size of database, number of records, nature of records, and nature of the data
- File type and format

c) Input parameters

- All parameters used to create the new database or resource

d) Output of the process

- Types of search queries supported by the database or resource
- Process of the query procedure

- Output data types and formats
- Application programming interfaces that can be used to access the resource

e) Regression testing, performance and scalability

- Description of the training and test datasets to check the validity of the claims made by the database or resource
- Description of the regression test process to verify the claims made by the database or resource

f) Provenance

- All metadata regarding the provenance of the database or resource and where they are accessible for independent enquiry, audit, review, assessment, verification and validation.

g) BioDBcore compliance

- All MIABi-DR databases must comply with the BioDBcore checklists and requirements, insofar as they do not conflict with the above specifications. Where there is a conflict, effort will be made to harmonise BioDBcore with MIABi-DR.

MIABi-SW (Software)

Scope – MIABi-SW is the Minimum Information About a Bioinformatics SoftWare which defines the minimum information reported for a new bioinformatics software.

Purpose – MIABi-SW contains information that can allow anyone suitably skilled in the art,

a) to reproduce the software with some effort (except in the case of a proprietary software - see below) and/or

b) to use the software, for the purposes of

i) determining the veracity of the claims of the authors of such a software found in the scientific publication that cites its compliance, and
ii) verifying the validity of the use of the software for the purposes of the scientific investigation citing its MIABi-compliant use, and
iii) reproducing the results which the publication that cites its compliance adduces.

Protection of Intellectual Property – Where a software is not intended for open source or open access, and that its content, though described as far as possible in compliance to MIABi, is classified proprietary and not revealed, then the MI disclosure shall include full reference to the source of these proprietary material and the mechanism for access or licensing or purchase, as the case may be, in order that the claims made by the publication can be verified or validated.

Content – MIABi-SW shall contain the following minimal information:

a) General descriptions

- Date stamp (as YYYY-MM-DD)
- Responsible person (or institutional role if more appropriate); provide name, affiliation and stable contact information
- Type of software
- Description of its functionalities
- Platforms supported
- For all external software and algorithms used in creating the software, the name, version and manufacturer
- Customizations made to that external software and algorithm
- Description of all internal software and algorithms used in creating the Software

- Availability of that external/internal software and algorithm
- Accessibility of the software, including mechanism of access (e.g. Internet)
- Nature of the accessibility (open source, freeware, shareware, crippled code, licensable software etc)
- Description of where the software is deposited and in what form (binaries, source code)
- Data transformation and normalization technique
- Overall research process, workflow pipelines and data flow diagrams where appropriate

b) Input data

- File type and format
- Datasets used (to be deposited in compliance with MIABi-DR)

c) Input parameters

- All parameters used to create the new software to derive the claims made by the final results

d) Output of the process

- Output data types and formats
- Sequence (for identified nucleotides, peptides, or proteins)
- Quantitative scores (for identified nucleotides, peptides, proteins, pathways, etc)
- Test output (to be deposited in compliance with MIABI-DR)

e) Regression testing, performance and scalability

- Description of the training and test datasets to check the validity of the claims made by the software

- Description of the regression test process to verify the claims made by the software

f) Provenance

- All metadata regarding the provenance of the software and where they are accessible for independent enquiry, audit, review, assessment, verification and validation.

MIABi-WS (Web Server)

Scope – MIABi-WS is the Minimum Information About a Bioinformatics Web Server which defines the minimum information reported for a new bioinformatics Web server.

Purpose – MIABi-WS contains information that can allow anyone suitably skilled in the art,

a) to reproduce the Web server with some effort (except in the case of a proprietary Web server - see below) and/or

b) to use the Web server, for the purposes of

iv) determining the veracity of the claims of the authors of such a Web server found in the scientific publication that cites its compliance, and

v) verifying the validity the web server for the purposes of the scientific investigation citing its MIABi-compliant use, and

vi) reproducing the results which the publication that cites its compliance adduces.

Protection of Intellectual Property – Where a Web server is not intended for open source or open access, and that its content, though described as far as possible in compliance to MIABi, is classified proprietary and not revealed, then the MI disclosure shall include full reference to the source of these proprietary material and

the mechanism for access or licensing or purchase, as the case may be, in order that the claims made by the publication can be verified or validated.

Content – MIABi-WS shall contain the following minimal information:

a) General descriptions

- Date stamp (as YYYY-MM-DD)
- Responsible person (or institutional role if more appropriate); provide name, affiliation and stable contact information
- Type of web server
- Description of its functionalities
- Platforms supported
- For all software and algorithms used in creating the Web server, the name, version and manufacturer
- Customizations made to that software and algorithm
- Availability of that software and algorithm
- Accessibility of the Web server, including mechanism of access (e.g. Internet)
- Data transformation and normalization technique
- Frequency of updates
- Overall research process, workflow pipelines and data flow diagrams where appropriate

b) Input data

- File type and format

c) Input parameters

- All parameters used to create the new Web server to derive the claims made by the final results

d) Output of the process

- Types of search queries supported by the Web server, where applicable
- Process of the query procedure
- Output data types and formats
- Sequence (for identified nucleotides, peptides, or proteins)
- Quantitative scores (for identified nucleotides, peptides, proteins, pathways, etc)

e) Regression testing, performance and scalability

- Description of the training and test datasets to check the validity of the claims made by the Web server
- Description of the regression test process to verify the claims made by the Web server

f) Provenance

- All metadata regarding the provenance of the web server and where they are accessible for independent enquiry, audit, review, assessment, verification and validation.
